# Malware Analysis Using Visualized Image Matrices

**DOI:** 10.1155/2014/132713

**Published:** 2014-07-16

**Authors:** KyoungSoo Han, BooJoong Kang, Eul Gyu Im

**Affiliations:** ^1^Department of Computer and Software, Hanyang University, Seoul 133-791, Republic of Korea; ^2^Department of Electronics and Computer Engineering, Hanyang University, Seoul 133-791, Republic of Korea; ^3^Division of Computer Science and Engineering, Hanyang University, Seoul 133-791, Republic of Korea

## Abstract

This paper proposes a novel malware visual analysis method that contains not only a visualization method to convert binary files into images, but also a similarity calculation method between these images. The proposed method generates RGB-colored pixels on image matrices using the opcode sequences extracted from malware samples and calculates the similarities for the image matrices. Particularly, our proposed methods are available for packed malware samples by applying them to the execution traces extracted through dynamic analysis. When the images are generated, we can reduce the overheads by extracting the opcode sequences only from the blocks that include the instructions related to staple behaviors such as functions and application programming interface (API) calls. In addition, we propose a technique that generates a representative image for each malware family in order to reduce the number of comparisons for the classification of unknown samples and the colored pixel information in the image matrices is used to calculate the similarities between the images. Our experimental results show that the image matrices of malware can effectively be used to classify malware families both statically and dynamically with accuracy of 0.9896 and 0.9732, respectively.

## 1. Introduction

Malware authors have been generating new malware and malware variants through various means, such as reusing modules or using automated malware generation tools. As some modules for malicious behavior are reused in malware variants, malware variants of the same family may have similar binary patterns, and these patterns can be used to detect malware and to classify malware families. Moreover, most antivirus programs focus on malware signatures, that is, string patterns, to detect malware [1]. However, various detection avoidance techniques such as obfuscation or packing techniques are applied to malware variants to avoid detection by signature-based antivirus programs and to make analysis difficult for security analysts [2, 3]. With the help of malware generation techniques, the amount of malware is increasing every year.

Although security analysts and researchers have been studying various analysis techniques to deal with malware variants, they cannot be analyzed completely because the malware in which avoidance techniques are applied is exponentially increasing. Therefore, new malware analysis techniques are required to reduce the burden on security analysts. Recently, several malware visualization techniques have been proposed to help security analysts to analyze malware.

In this paper, we propose a novel method to analyze malware visually to classify malware families. The proposed method converts the opcode sequences extracted from the malware into images called image matrices and calculates the similarities between each image. In addition, we apply the proposed method to the execution traces extracted through dynamic analysis, so that malware employing detection avoidance techniques such as obfuscation and packing can be analyzed. To reduce the computational overheads, we extract the opcode sequences only from the blocks that are related to staple behaviors, such as functions and application programming interface (API) calls, by using a major block selection technique [2]. Representative images of individual malware families are generated and are used to classify the unknown sample rapidly. Using these image matrices, we obtain the similarities between the images after the RGB-colored pixel information of the images is vectorized and the pixel similarities are calculated.

This paper is composed as follows. In Section 2, malware analysis-related studies are described. In Section 3, malware analysis methods using visualized opcode sequences and the methods to calculate similarity are proposed, and the experimental results are presented in Section 4. Finally, in Section 5, conclusions and future directions are provided.

## 2. Related Work

In general, malware analysis methods to detect and classify malware can be categorized as either static or dynamic analyses [4]. In the static analysis of malware, various methods such as control flow graph (CFG) analysis [5–7], call graph analysis [8, 9], byte level analysis [10], instruction-based analysis [11–14], and similarity-based analysis [15, 16] have been proposed.

CFGs are generated by dividing the instructions extracted through disassembling into blocks and by connecting the directed edges between the blocks. Some malware analysis methods using these CFGs as signatures have been proposed. Cesare and Xiang [5] proposed a method that defines CFGs as signatures in string form that consist of a list of graph edges for the ordered nodes and that measures the similarities among signatures by using the Dice coefficient algorithm [17]. Bonfante et al. [6] proposed a method that converts the CFGs into tree-based finite state machines through syntactic analysis and semantic analysis and then uses them as signatures. Briones and Gomez [7] proposed an automated classification system based on CFGs. The CFGs are summarized as three tuples including the number of basic blocks, the number of edges, and the number of subcalls, and then two functions can be compared. However, if the complex information is summarized into a small size, high false alarms may occur.

There is much research aimed at detecting malware based on information such as system-calls, functions, and API calls, which is used for malware execution in operating systems. Shang et al. [8] proposed a method that generates function-call graphs, which represent the caller and callee relationships between functions as signatures of malware samples, and they then compute the similarities by using those function-call graph signatures. Kinable and Kostakis [9] classified malware using the call graph clustering technique. Their proposed method generated the call graphs against the functions included in the malware samples, and they performed the clustering based on the structural similarity scores of the call graphs calculated through the graph edit distance algorithm.

Statistical information regarding the instructions extracted through disassembling can be used in the static analysis of malware. Rad and Masrom [11] proposed a method based on the instruction frequencies in order to classify metamorphic malware. Since instruction frequencies are mostly not changed, even though the obfuscation techniques are applied to the malware, the instruction frequencies can become the features of malware. Therefore, their proposed method calculated a distance by using the instruction frequencies extracted from each malware sample, and they then classified metamorphic malware by using the distance value. Bilar [12] showed that there were different instruction frequencies in different malware. Particularly, they showed that rare instructions in malware could become better predictors to classify malware than other instruction could. Han et al. [13] proposed a method using instruction frequencies. The proposed method generated instruction sequences that were sorted according to the instruction frequencies, and they showed that the distances between instruction sequences from the same malware family had low distance values. Santos et al. [14] proposed a malware classification method using* n*-gram instruction frequencies in which* n*-gram instructions included* n*-instructions. In the proposed method, they generated the vectors for each n-instruction sequence and used some of the vectors as signatures.

In addition, dynamic analysis methods including tainting, behavior-based methods, and API call monitoring have been proposed. Egele et al. [18] proposed a method using tainting techniques, which tracks the behaviors related to the flow of information that are processed by any browser helper object (BHO). If the BHO leaks sensitive information to the outside, the BHO is classified as malware. Fredrikson et al. [19] proposed a method that automatically extracts the characteristics of behaviors by using graph mining techniques. Their proposed method made clusters by identifying core CFGs for each similar malicious behavior in a malware family, and these were then generalized as a significant behavior. Furthermore, methods based on dynamic monitoring techniques using an emulator have been proposed. Vinod et al. [20] traced malware API calls via dynamic monitoring within an emulator and measured their frequencies to extract critical APIs. Miao et al. [21] developed a tool called the “API Capture” that extracts the major characteristics automatically, such as system-call arguments, return values, and error conditions by monitoring malware behavior in an emulator.

Even though there are many static and dynamic analyses methods available, new techniques that can complement existing techniques are still needed to improve malware analysis performance and conveniences of analysis by security analysts. Recently, several visualization methods have been proposed to help security analysts to observe the features and behaviors of malware [22]. To visualize malware behavior, Trinius et al. [23] proposed a method that visualized the percentages of API calls as well as malware behavior into each of two images called a “treemap” and “thread graph,” respectively. Saxe et al. [24] developed a system that generated two types of images. One image showed the system-call sequences extracted from malware system-call behavior logs, and the other image showed similarities and differences between selected samples. Conti et al. [25] proposed a visualizing system that shows the images for the byte information of malware samples such as byte values, byte presence, and duplicated sequences of bytes contained within a sample. Anderson et al. [26] proposed a method to show the similarities between malware samples in an image named a “heatmap.” Nataraj et al. [27] converted the byte information into gray-scale images and classified the malware using image processing. After generating images using byte values, they applied an abstract representation technique for the scene image, that is, GIST [28, 29], to compute texture features. Moreover, they proved that the binary texture analysis techniques using image processing could classify malware more quickly than existing malware classification methods could [30]. However, since the texture analysis method has large computational overheads, the proposed method has problems in processing a large amount of malware [31].

In this paper, we propose a novel analysis method using image matrices to represent malware visually so that the features of the malware can be easily detected and the similarities between different malware samples can be calculated faster than with other visualization methods.

## 3. Our Proposed Method

### 3.1. Overview

**Figure 1:**
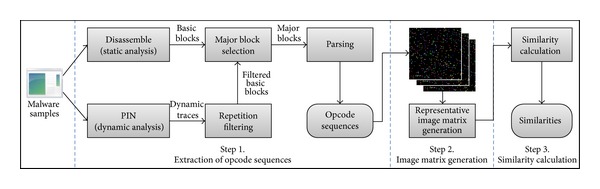
Overview of the proposed method.

Our proposed visualized malware analysis method consists of three steps, as shown in Figure 1. In Step 1, opcode sequences are extracted from malware binary samples or dynamic execution traces. Then, image matrices in which the opcode sequences are recorded as RGB-colored pixels are generated in Step 2. In Step 3, the similarities between the image matrices are calculated. In the following sections, each step is explained in detail.

### 3.2. Extraction of Opcode Sequences

**Figure 2:**
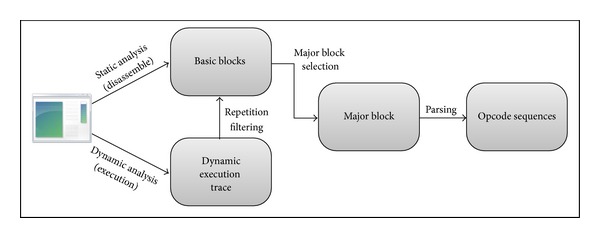
Opcode sequence extraction procedure.

Figure 2 shows the process to extract opcode sequences from malware binary samples for Step 1 through static analysis or dynamic analysis.

#### 3.2.1. Basic Block Extraction

To extract opcode sequences from malware binary samples, the binary sample files are first disassembled and divided into basic blocks, using disassembling tools, such as IDA Pro [32] or OllyDbg [33]. However, if obfuscation or packing techniques are applied in malware samples, static analysis using a disassembler is not feasible [34, 35]. Therefore, some malware samples (in which obfuscation or packing techniques are applied) need to be executed in a dynamic analysis environment [36].

**Figure 3:**
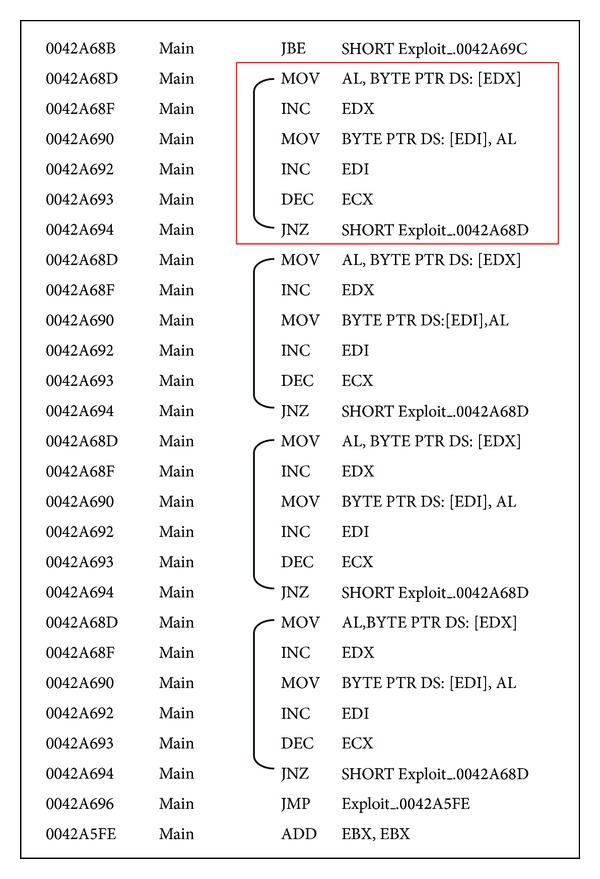
An example of repeated instruction sequences.

In dynamic analysis, as shown in Figure 3, some repeated instruction sequences are included in the dynamic execution traces because a program may have some loops or repeated calls, and these repeated sequences can increase the size of not only the execution traces, but also the processing overheads. Kang et al. [37] proposed a repetition filtering method for dynamic execution traces. Our filtered basic blocks are extracted from the dynamic execution traces after the repetition filtering method is applied. Finally, if basic blocks are extracted from malware samples or dynamic execution traces, then major blocks are selected from the basic blocks by our proposed technique, which is explained in the next section.

#### 3.2.2. Major Block Selection

The malware analysis method proposed in this paper does not target all of the basic blocks from the binary disassembling results or dynamic execution traces. If all the basic blocks are used for analysis, then some blocks for binary file execution in an operating system are included in the basic blocks. Moreover, many meaningless blocks may be included in the basic blocks extracted from malware samples. As a result, the number of basic blocks that have to be analyzed by the security analysts is increased and distinguishing malware features becomes difficult. In addition, the number of comparisons between the basic blocks from two malware samples is also increased dramatically. On the contrary, if the number of unnecessary blocks can be reduced as much as possible in the malware analysis, the analysis time cost for not only the individual malware sample, but also a large number of malware samples can be reduced. Therefore, we selected some blocks relating to suspicious behaviors and functions from among the entire set of basic blocks.

**Figure 4:**
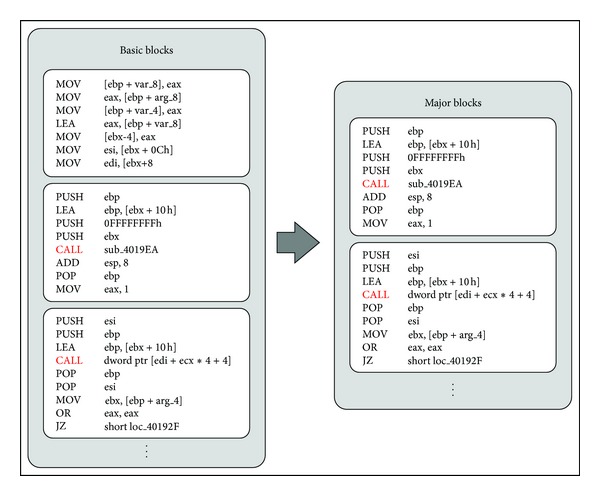
Major block selection.

As shown in Figure 4, the blocks selected as major blocks are those that include the* CALL* instruction, which is used to invoke APIs, library functions, and other user-defined functions. This is because not only user-defined functions, but also various system calls are used to implement the behaviors and functions of most programs. If blocks that include these function invocation instructions are used in malware analysis, malware features can be extracted [2]. Through a major block selection technique, the image matrix generating time is reduced by recording only those selected blocks in the image matrix.

#### 3.2.3. Opcode Sequence Extraction

**Figure 5:**
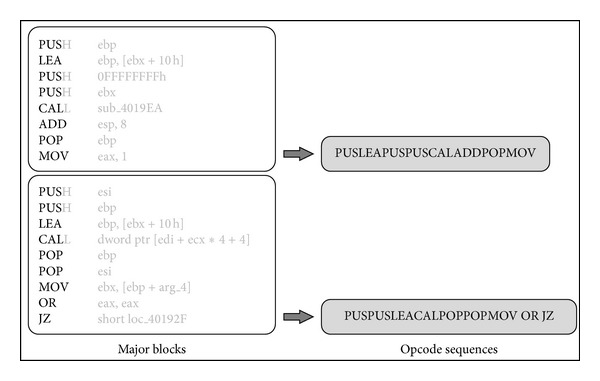
Opcode sequences used as malware information.

To extract malware features, as shown in Figure 5, the opcode sequences in the individual major blocks are used as malware information. From each opcode, only the first three characters are used to generate information for the block. The reasons for using a three-character opcode are as follows. From the entire set of opcodes used in the Intel x86 assembly language, 41.4% of them have three characters, and the appearance frequencies of these opcodes within the binary files are higher than for other opcodes. On the other hand, 28.8% of opcodes have four characters, 17.8% have five characters, and 5.2% have over six characters, respectively; thus, their appearance frequencies are relatively low. In addition, since the meanings of the individual opcodes are maintained even though they are reduced to three characters, the different opcodes can be distinguished. For example, four-character opcodes such as* PUSH* are reduced to three characters,* PUS*, and two-character opcodes such as* OR* are expanded by adding a blank character to a three-character opcode. Then, these three-character opcodes are concatenated together, and the character string is used to represent the block as an opcode sequence, which is used to generate image pixels in an image matrix in the next step.

### 3.3. Generation of the Image Matrix

**Figure 6:**
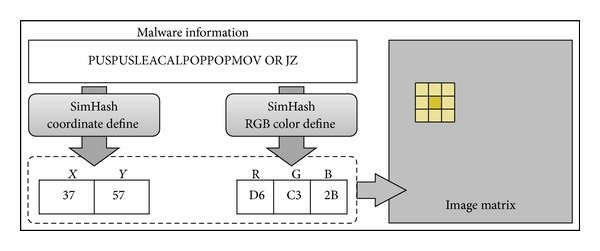
Generating images using opcode sequences.

Figure 6 shows the procedure for Step 2 that converts the opcode sequences into pixels in an image matrix. A hash function is used to decide the *X*-*Y* coordinates and RGB colors of the pixels.

To visualize a binary file as an image matrix, both the length and the width of an image matrix are initialized to 2^*n*^, where *n* is selected by the users. To reduce the probability of collisions of the hash function;* n* should be large enough. In our experiments, we selected *n* as 8 to minimize collisions.

The coordinate-defining module and the RGB color-defining module are used to generate image matrices. First, the coordinate-defining module defines the (*x*, *y*) coordinates of pixels on the image matrix of each code block. Second, the RGB color-defining module defines the color values of pixels on the image matrix. RGB colors are defined by calculating values of 8 bits each for the red, green, and blue colors.

*SimHash* [38] is applied to opcode sequences extracted in Step 1 in order to define both the coordinates and the color values of the pixels.* SimHash* is a local-sensitive hash function used in the similar sentence detection system, which assumes that if the input values are similar, then the output values will also be similar. That is, since* SimHash* tokenizes the input strings and generates hash values for each token, if a few tokens are different in two input strings, then the generated hash values are not completely different, but are similar. Therefore, if the character strings of the opcode sequences are similar, then the outputs will be similar, and they will map onto similar coordinates in an image matrix.

**Figure 7:**
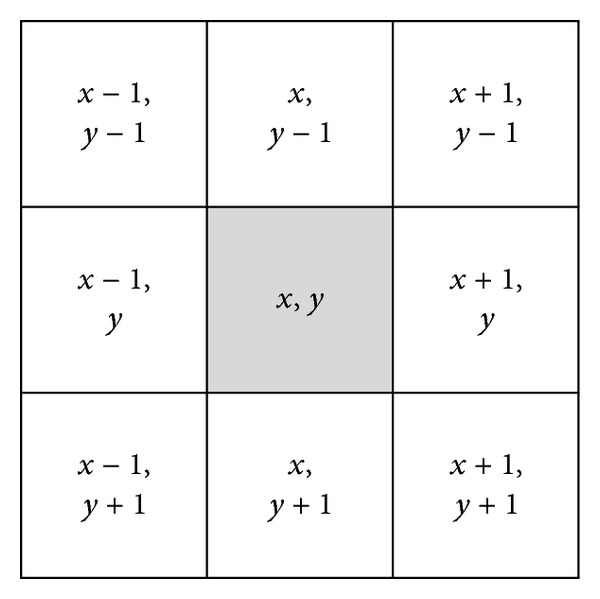
Nine pixels for one opcode sequence.

Once the coordinates and RGB colors of the individual pixels have been defined, RGB-colored images are recorded on the individual coordinates of image matrices. To provide human analysts with a more convenient visual analysis, pixels around the defined coordinates are recorded simultaneously. As shown in Figure 7, nine pixels from (*x* − 1, *y* − 1) to (*x* + 1, *y* + 1) around an (*x*, *y*) coordinate for a block are recorded.

**Figure 8:**
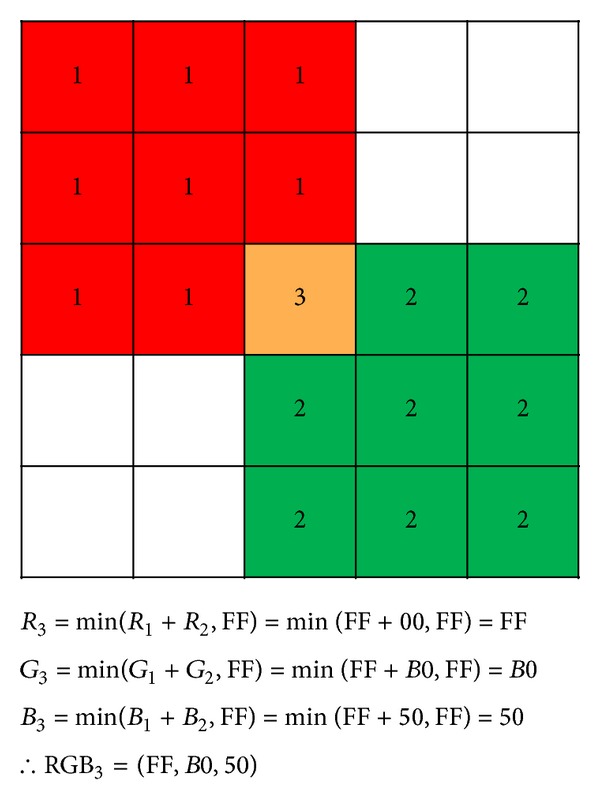
Method of recording overlapping pixels.

If the images overlap each other because the coordinates defined for multiple opcode sequences are adjacent, as shown in Figure 8, the sums of RGB colors become new pixel colors. If the result of a color summing exceeds 255 (0xFF), the result will be set to 255. For example, if RGB_1_ is (255,0, 0) and RGB_2_ is (0,176,50), the new color will become (255,176,50).

The number of pixels recorded on an image matrix varies according to the major blocks, and the number of overlapping pixels will increase as the number of images increases. If there are too many overlapping images, then the size of the image matrix should be increased.

### 3.4. Representative Image Matrix Extraction

Since many malware variants exist in each malware family, as the number of malware samples increases, the total amount and time of the similarity calculation increase, too. Therefore, we extracted a representative image matrix of each malware family to reduce the costs of malware similarity calculations. That is, when a new malware sample is found, the amount of time to calculate the similarity is reduced by comparing the image matrix of the new malware with the image matrices that represent individual malware families instead of comparing it with all of the image matrices of the existing malware samples.

**Figure 9:**
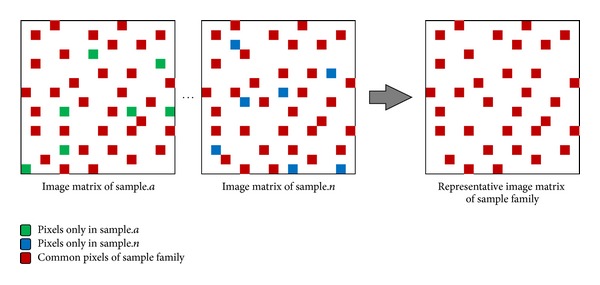
Representative image matrix extraction.

As shown in Figure 9, to extract a representative image matrix for each malware family, image matrices are generated for samples in malware families. Then, the representative image matrix is extracted by recording only the common pixels that have same coordinates and RGB colors from the image matrices of individual malware samples in the same family. Figure 9 shows an example of the generation of representative images of malware families.

### 3.5. Similarity Calculation Using Image Matrices

The advantage of the similarity calculation using the image matrices is a faster performance than with exact matching using the string type of opcode sequences, even though there are some extra false positives due to hash collision. When using the string, the time complexity is defined as *O*(*n* ^2^) due to the process of finding pairs of exactly matched strings. However, if the image matrices are used to calculate similarities, since the coordinates and colors of the opcode sequences are defined through* SimHash*, the process of finding the pairs is skipped. Therefore, the time complexity of the similarity calculations using the image matrices is defined as* O*(*n*), because only the color information of the pixels recorded on the same coordinates in both image matrices is used to calculate the similarities between the image matrices.

**Figure 10:**
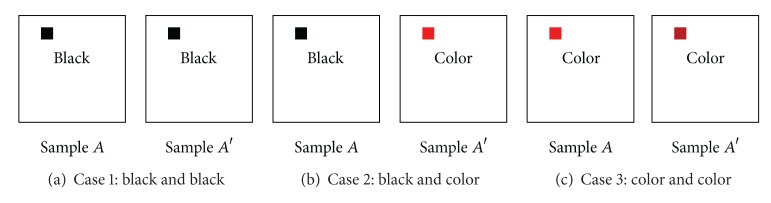
Three cases considered in pixel similarity calculations.

Pixel similarity calculations are carried out first for pixels in each image matrix. The most important consideration in a similarity calculation in this case is that only those RGB color pixels recorded in the individual image matrices should be used. Image matrices have RGB-colored pixels on square images with black backgrounds. If black pixels are also used in similarity calculations, the similarities between samples from different malware families can be calculated as very high. Therefore, when the similarities of the image matrices are calculated, the following cases are considered for pixels on the same coordinates in the two image matrices, as shown in Figure 10. In this case, the vector angular-based distance measurement algorithm is used to calculate the similarities between color pixels. This algorithm calculates similarity values by expressing the color pixels constituting each image as 3D vectors, as shown in (1), and then using the angle information and size information

(a) Case 1: if all of the pixels in the areas of both image matrices are black, the pixel similarity calculation will not be carried out and the next pixel will be selected.
(b) Case 2: if one pixel in a selected area is black and the corresponding pixel in the other image is colored, the pixel similarity will be defined as 0.
(c) Case 3: if both pixels are not black but colored, the color pixel similarity will be calculated using the vector angular-based distance measurement algorithm [39], as follows:
  (1) $$\begin{array}{c} \delta \left(x_{i},x_{j}\right)=\left[1-\frac{2}{\pi }\cos^{-1}\left(\frac{x_{i}\cdot x_{j}}{\left|x_{i}\right|\left|x_{j}\right|}\right)\right]\left[1-\frac{\left|x_{i}-x_{j}\right|}{\sqrt{3\cdot 255^{2}}}\right]. \end{array}$$

The similarity values of the image matrix when considering individual cases are calculated, using the results from the pixel similarity calculations, as shown in (2). That is, the sum of pixel similarity values calculated in case 3 is divided by the number of pixels calculated in cases 2 and 3 to calculate the average:

(2) $$\begin{array}{c} \text{Sim}\left(A,B\right)=\frac{\text{sum}\;\text{of}\;\text{pixel}\;\text{similarity}\;\text{values}\;\text{in}\;\text{case}\;3}{\#\;\text{of}\;\text{pixels}\;\text{in}\;\text{case}\;2\;\text{and}\;\text{case}\;3}. \end{array}$$

## 4. Experimental Results

### 4.1. Experimental Data and Environment

Using the visual analysis tools implemented in this paper, and the malware samples shown in Table 1, image matrices were generated, and similarity calculations were performed. First, set A consists of 290 malware samples from 16 families in which the detection avoidance techniques, such as obfuscation and packing, are not applied. These malware samples are used to extract the basic blocks through static analysis using a disassembler. Second, set B consists of 560 malware samples from 14 families in which the packed and nonpacked malware samples coexist. We used these malware samples to generate dynamic execution traces through the PIN tool in a dynamic analysis environment, and the filtered basic blocks are extracted from the dynamic execution traces through the repetition filtering technique, as explained in Section 3.2.1.

**Figure 11:**
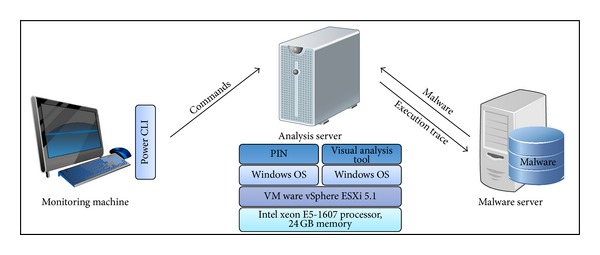
Experimental environment.

For the experiments, we constructed an experimental environment consisting of the analysis server, malware server, and monitoring machine, as shown Figure 11. We set up VMware vSphere ESXi 5.1 in the analysis server, which has an Intel Xeon E5-1607 processor and 24 GB of main memory, and we installed two Windows operating systems (OSs) as guest OSs. In the first Windows OS, the dynamic execution traces were extracted through the PIN. In the other Windows OS, the image matrices were generated and similarities were calculated through our visual analysis tool. Malware samples that are provided for the analysis server and the dynamic execution traces extracted from the analysis server are stored in the malware server. The monitoring machine controls the analysis server through the PowerCLI tool that is the remote command line interface.

### 4.2. Experiments with Static Analysis

For the experiments in this section, we disassembled the malware samples within set A and extracted major blocks from the basic blocks. We then generated the image matrices using opcode sequences of those major blocks and analyzed the similarities among them.

#### 4.2.1. Image Matrix Generation

In this paper, we set the sizes of the generated image matrices to 256 × 256 pixels for the experiments. As shown in Table 2, the reasons for using this image matrix size can be briefly summarized as the middle ground between file size, similarity calculation time, and classification accuracy. The accuracy was calculated by using (3):

(3) $$\begin{array}{c} \text{Accuracy}=\frac{\#\;\text{of}\;\text{correctly}\;\text{classified}\;\text{malware}\;\text{samples}}{\#\;\text{of}\;\text{total}\;\text{malware}\;\text{samples}}. \end{array}$$

**Figure 12:**
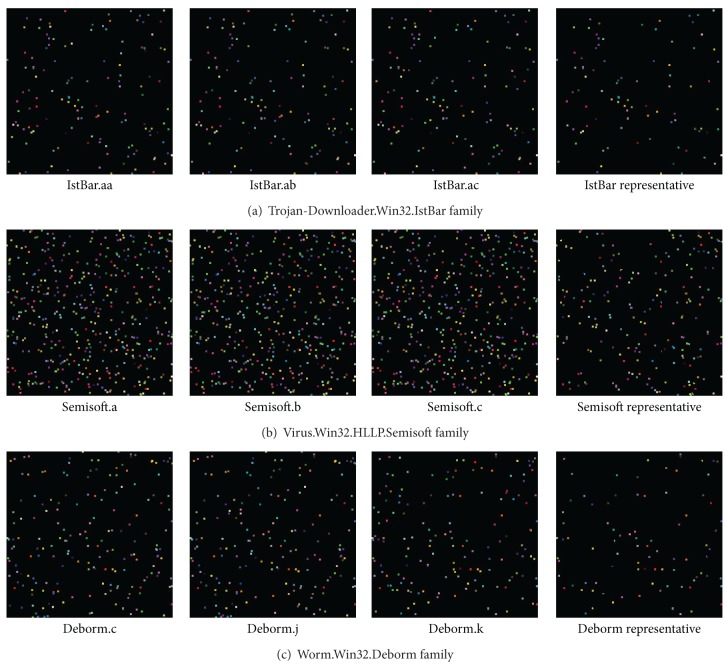
Image matrices of malware family samples.

Figure 12 shows examples of the image matrices generated from the malware samples of individual families within set A. Only three image matrices for each malware family and one representative image matrix extracted by recording only those pixels commonly existing in all image matrices were included. Since the number of opcode sequences used as malware information varied, the number of pixels recorded on the image matrices differed. In the case of malware, many of the same or similar RGB-colored pixels are found among the image matrices of malware samples classified as the same family. However, even if pixels are recorded on the same coordinates of different image matrices, the pixel similarities have different values if the RGB color information of the relevant pixels is different. Our results show that image matrices of variants included in the same malware family can be shown to be similar and that clear differences exist among malware samples from different families.

**Figure 13:**
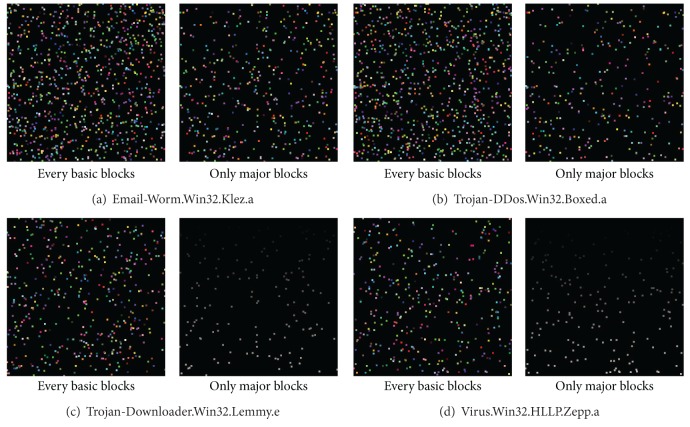
Comparison of image matrices with and without major block selection.

Figure 13 shows the image matrix differences before and after the application of the major block selection technique. The image progression indicates that the number of pixels recorded in the image matrices decreases because of the selection of major blocks from among the basic blocks. The similarity changes after the application of the major block selection is described in the next subsection.

#### 4.2.2. Major Block Selection

**Figure 14:**
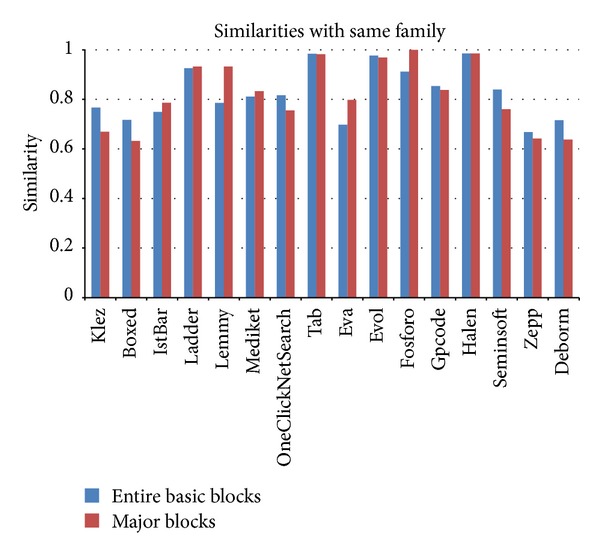
Image matrix similarity calculations of malware samples in each family following major block selection.

**Figure 15:**
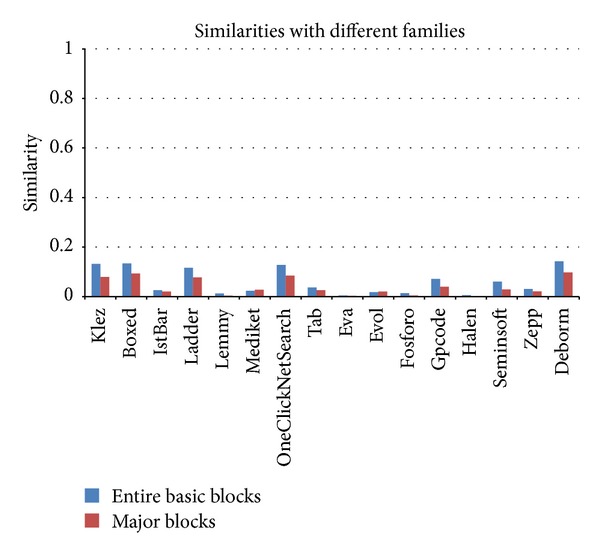
Image matrix similarity calculations of malware samples from different families following major block selection.

Similarity calculations of the image matrices after the application of major block selection are shown in Figures 14 and 15. When the major block selection technique was applied, the similarity changes ranged from a minimum of 0.002 (the Tab family) to a maximum of 0.147 (the Lemmy family) among the malware samples in the same families. The results of the similarity calculations for different families showed that the changes ranged from a minimum of 0.001 (the Eva family) to a maximum of 0.053 (the Klez family). As a result, while the range of the similarity values among the malware samples in the same family is more than 0.6, the range of the similarity values among malware samples from different families is below 0.1. Therefore, although the similarity values change due to applying the major block selection technique, we can reduce the image matrix generation time and can find obvious differences in the similarity values.

#### 4.2.3. Representative Image Matrix Extraction

For this experiment, we selected an arbitrary malware sample not included in data set A as the unknown sample. We then analyzed the similarity calculation time and the similarity values of an image matrix for the unknown sample with 290 image matrices of all malware samples and with 16 representative image matrices extracted from individual families.

**Figure 16:**
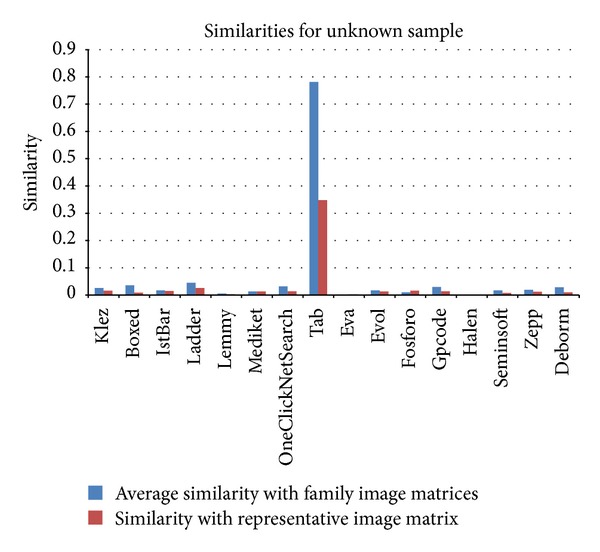
Similarity values of the unknown sample compared to the representative image matrices of individual families.

Figure 16 shows the results of the similarity calculations of an unknown sample both with all of the image matrices of malware samples and with the representative image matrices of individual families. When all of the image matrices were used, the Tab family was found to have an average similarity value of 0.781, while all the other families had values smaller than 0.05. When representative image matrices of individual families were used, the average similarity of the Tab family had a value of 0.348, while the other families had values of less than 0.03. Therefore, the unknown sample is expected to be a variant of the Tab family. In fact, the diagnostic name of the unknown sample used for this experiment was Trojan-Dropper. Win32.Tab.gd.

Table 3 shows the list of the malware samples selected as unknown samples for this experiment and it includes the results of the similarity calculations using representative image matrices. These malware samples except for Agobot.02.a and Sdbot.04.a were detected as variants of each corresponding family in set A, with a range of similarity values among the representative image matrices from 0.181 to 0.352. Since the Agobot.02.a and the Sdbot.04.a samples are not variants of the malware families in set A, their similarity values compared to the existing individual family representative image matrices were very low.

#### 4.2.4. Feasibility in Malware Classification

**Figure 17:**
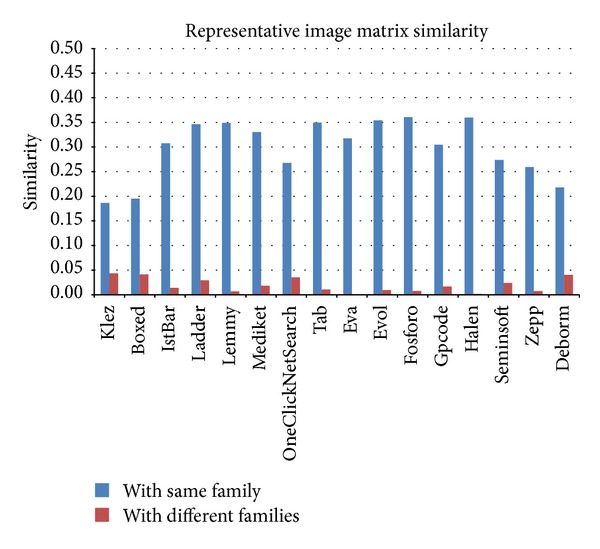
Results of similarity calculations using the three proposed techniques.

Figure 17 shows the changes in similarity values obtained by applying all the proposed methods together, that is, the major block selection and representative image extraction techniques. Whereas the similarities between malware samples from the same families had values between 0.19 and 0.36, the similarities between malware samples from different families were less than 0.05. The classification accuracy, which was obtained by using the image matrices that were generated through the static analysis, was 0.9896. That is, only three malware samples in set A were misclassified into the other malware families. Our result was a little better than the average classification accuracy of 0.9757 using the binary texture analysis in [30]. Therefore, we conclude that our methods are feasible for malware classification because similarities within the same families will be relatively high compared to the similarities between malware samples from different families.

### 4.3. Execution Trace-Based Experiments

For the execution trace-based experiments, the malware samples within set B in Table 1 were executed in dynamic analysis environments using the PIN tool. Dynamic execution traces were then generated, and the repetition-filtered basic blocks were extracted from those execution traces. After filtering, the major blocks relating to suspicious behaviors and functions were selected. Our proposed techniques were applied to these execution traces to generate image matrices and to analyze similarities.

**Figure 18:**
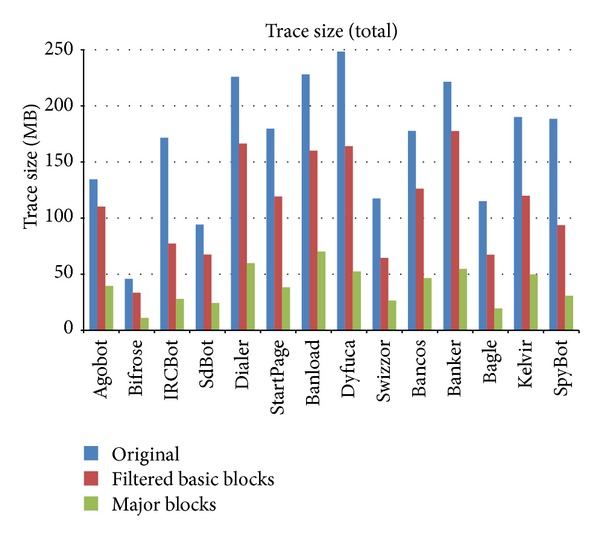
Changes in execution trace size following the application of repetition filtering and major block selection.

Figure 18 shows the decrease in size of the execution traces resulting from the application of the repetition filtering and major block selection techniques. If the sizes of the execution traces were first reduced through the repetition filtering technique and then the major block selection method was applied, the sizes of the execution traces were reduced by 76.5% on average (69.3% minimum, 83.6% maximum) compared to the original execution traces.

**Figure 19:**
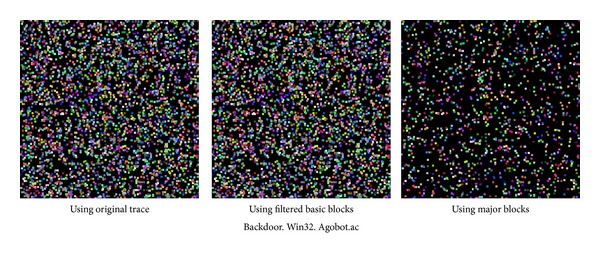
Changes in the generated image matrices from the application of repetition filtering and major block selection.

Figure 19 shows changes in the generated image matrices resulting from the application of the repetition filtering method and the major block selection. Decreases in the number of recorded pixels in the image matrices can be recognized when the three image matrices are compared.

**Figure 20:**
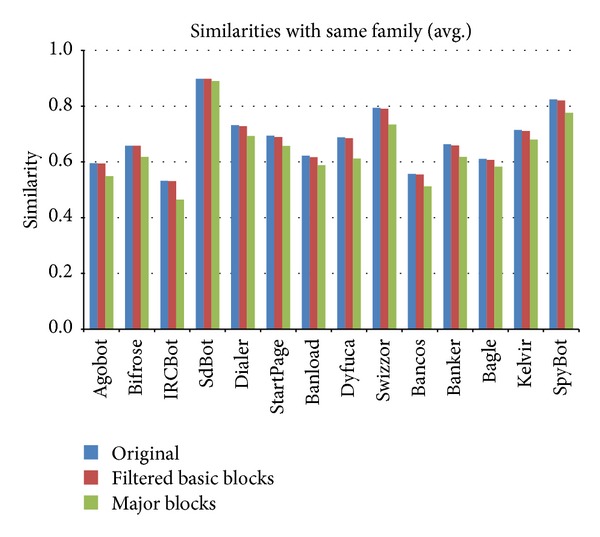
Changes in similarity between samples in the same families after repetition filtering and major block selection.

**Figure 21:**
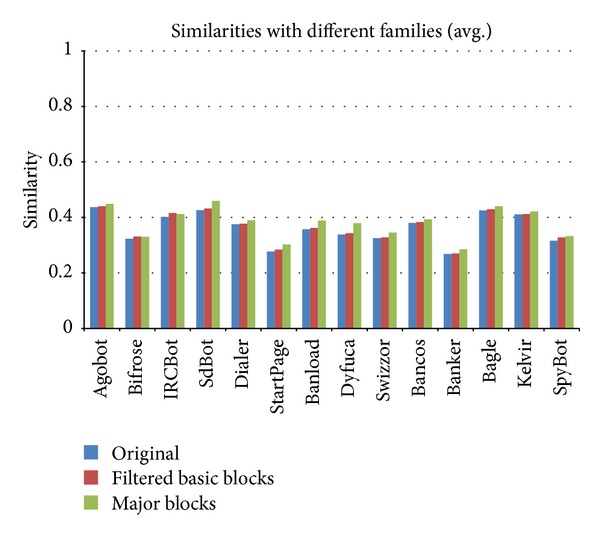
Changes in similarity between samples from different families after repetition filtering and major block selection.

Figures 20 and 21 show changes in the similarity values with the application of the repetition filtering technique and the major block selection. Although changes in the values are not large, some malware families are distinguishable if the threshold of similarity values is set properly.

In these experiments, the average similarity values of malware samples from the same families was approximately 0.65 and those from different families were approximately 0.36. Compared to the results of the static analysis described previously, the results from the execution trace-based experiments show relatively small differences. The reason for these results is that similar system dynamic link libraries (DLLs) were invoked when the malware samples of each family were executed in the dynamic analysis environment to extract the dynamic execution traces. As a result, similar opcode sequences due to the DLL calls and the executing of DLLs from the dynamic execution traces were recorded in the image matrices of individual families, so the similarity values increased. Nevertheless, the classification accuracy obtained through the similarity calculations using the image matrices that were generated based on the execution traces was 0.9732 because only 15 malware samples in set B were misclassified, and this result was similar to the accuracy in [30].

## 5. Conclusions and Future Work

In this paper, we proposed a novel method to analyze malware samples visually by generating image matrices. To generate the image matrices, opcode sequences were extracted through static analysis and dynamic analysis. In addition, we calculated the similarities between the malware variants using vectorized values of the RGB-colored pixels in the image matrices. The similarity calculation method using the image matrices has a faster performance than exact matching using the string type of opcode sequences or basic blocks. Our proposed method was implemented as a visual analysis tool. The experimental results showed that malware variants included in the same family were similar when converted into image matrices, and the similarities between malware variants were shown to be higher. With our proposed method, security analysts can analyze malware samples visually and can distinguish similar malware samples for further analysis. Our future studies include faster malware detection and classification using the parallelization techniques and real-time processing based on GPGPU.

## Figures and Tables

**Table 1:** Malware samples.

Set	Type	Family	Number of variants
A	Email-Worm	Klez	9
	Trojan-DDos	Boxed	27
	Trojan-Downloader	IstBar	41
		Ladder	5
		Lemmy	26
		Mediket	43
		OneClickNetSearch	11
	Trojan-Dropper	Tab	8
	Virus	Eva	6
		Evol	3
		Fosforo	4
		Gpcode	35
		Halen	7
		Semisoft	14
		Zepp	11
	Worm	Deborm	40

B	Backdoor	Agobot	40
		Bifrose	40
		IRCBot	40
		SdBot	40
	Trojan	Dialer	40
		StartPage	40
	Trojan-Downloader	Banload	40
		Dyfuca	40
		Swizzor	40
	Trojan-Spy	Bancos	40
		Banker	40
	Email-Worm	Bagle	40
	IM-Worm	Kelvir	40
	P2P-Worm	SpyBot	40

**Table 2:** The selection of image matrix size.

Size (resolution)	File size (KB)	Similarity calculation time (ms, avg.)	Classification accuracy (avg.)
128 × 128	48	5.3	0.9595
256 × 256	*192 *	*18.2 *	*0.9814 *
512 × 512	768	66.4	0.9929

**Table 3:** Arbitrarily selected malware samples as unknown.

Number	Malware sample	Most similar family (similarity)	
1	Klez.j	Klez	(0.181)
2	Boxed.g	Boxed	(0.190)
3	IstBar.gvf	IstBar	(0.302)
4	Ladder.f	Ladder	(0.339)
5	Lemmy.z	Lemmy	(0.341)
6	Mediket.ec	Mediket	(0.325)
7	OneClickNetSearch.k	OneClickNetSearch	(0.268)
8	Tab.gd	Tab	(0.348)
9	Eva.g	Eva	(0.317)
10	Evol.c	Evol	(0.329)
11	Fosforo.d	Fosforo	(0.341)
12	Gpcode.x	Gpcode	(0.306)
13	Halen.2619	Halen	(0.352)
14	Semisoft.n	Semisoft	(0.281)
15	Zepp.d	Zepp	(0.265)
16	Deborm.ai	Deborm	(0.339)
17	Agobot.02.a	Mediket	(0.042)
18	SdBot.04.a	Boxed	(0.054)

## References

[1] Christodorescu M, Jha S Testing malware detectors.

[2] Kang B, Kim T, Kwon H, Choi Y, Im EG Malware classification method via binary content comparison.

[3] Moser A, Kruegel C, Kirda E Limits of static analysis for malware detection.

[4] Ernst MD Static and dynamic analysis: synergy and duality.

[5] Cesare S, Xiang Y A fast flowgraph based classification system for packed and polymorphic malware on the endhost.

[6] Bonfante G, Kaczmarek M, Marion J-Y (2009). Architecture of a morphological malware detector. *Journal in Computer Virology*.

[7] Briones I, Gomez A Graphs, entropy and grid computing: automatic comparison of malware. http://pandalabs.pandasecurity.com/blogs/images/PandaLabs/2008/10/07/IsmaelBriones-VB2008.pdf.

[8] Shang S, Zheng N, Xu J, Xu M, Zhang H Detecting malware variants via function-call graph similarity.

[9] Kinable J, Kostakis O (2011). Malware classification based on call graph clustering. *Journal in Computer Virology*.

[10] Tabish SM, Shafiq MZ, Farooq M Malware detection using statistical analysis of byte-level file content.

[11] Rad BB, Masrom M Metamorphic virus variants classification using opcode frequency histogram.

[12] Bilar D (2007). Opcodes as predictor for malware. *International Journal of Electronic Security and Digital Forensics*.

[13] Han KS, Kim S-R, Im EG (2012). Instruction frequency-based malware classification method. *Information*.

[14] Santos I, Brezo F, Nieves J (2010). Idea: Opcode-sequence-based malware detection. *Engineering Secure Software and Systems*.

[15] Sung AH, Xu J, Chavez P, Mukkamala S Static analyzer of vicious executables (SAVE).

[16] Walenstein A, Venable M, Hayes M, Thompson C, Lakhotia A Exploiting similarity between variants to defeat malware.

[17] Chowdhury G (2010). *Introduction to Modern Information Retrieval*.

[18] Egele M, Kruegel C, Kirda E, Yin H, Song DX Dynamic spyware analysis.

[19] Fredrikson M, Jha S, Christodorescu M, Sailer R, Yan X Synthesizing near-optimal malware specifications from suspicious behaviors.

[20] Vinod P, Jain H, Golecha YK, Gaur MS, Laxmi V Medusa: metamorphic malware dynamic analysis using signature from API.

[21] Miao QG, Wang Y, Cao Y, Zhang XG, Liu ZL APICapture—a tool for monitoring the behavior of malware.

[22] Han KS, Lim JH, Kang B, Im EG (2014). Malware analysis using visualized images and entropy graphs. *International Journal of Information Security*.

[23] Trinius P, Holz T, Göbel J, Freiling FC Visual analysis of malware behavior using treemaps and thread graphs.

[24] Saxe J, Mentis D, Greamo C Visualization of shared system call sequence relationships in large malware corpora.

[25] Conti G, Dean E, Sinda M, Sangster B (2008). Visual reverse engineering of binary and data files. *Visualization for Computer Security*.

[26] Anderson B, Storlie C, Lane T Improving malware classification: bridging the static/dynamic gap.

[27] Nataraj L, Karthikeyan S, Jacob G, Manjunath B Malware images: visualization and automatic classification.

[28] Oliva A, Torralba A (2001). Modeling the shape of the scene: a holistic representation of the spatial envelope. *International Journal of Computer Vision*.

[29] Torralba A, Murphy KP, Freeman WT, Rubin MA Context-based vision system for place and object recognition.

[30] Nataraj L, Yegneswaran V, Porras P, Zhang J A comparative assessment of malware classification using binary texture analysis and dynamic analysis.

[31] Kang Y, Sugimoto A (2014). Image categorization and semantic segmentation using scale-optimized textons. *Journal of IT Convergence Practice*.

[32] Eagle C (2008). *The IDA Pro Book: The Unofficial Guide to the World's Most Popular Disassembler*.

[33] Yuschuk O Ollydbg. http://www.ollydbg.de.

[34] Wei Y, Zheng Z, Ansari N (2008). Revealing packed malware. *IEEE Security and Privacy*.

[35] Royal P, Halpin M, Dagon D, Edmonds R, Lee W PolyUnpack: automating the hidden-code extraction of unpack-executing malware.

[36] Berkowits S Pin—A Dynamic Binary Instrumentation Tool. https://software.intel.com/en-us/articles/pin-a-dynamic-binary-instrumentation-tool.

[37] Kang B, Han KS, Kang B, Im EG (2013). Malware categorization using dynamic mnemonic frequency analysis with redundancy filtering.

[38] Charikar MS Similarity estimation techniques from rounding algorithms.

[39] Androutsos D, Plataniotis KN, Venetsanopoulos AN (1999). Novel vector-based approach to color image retrieval using a vector angular-based distance measure. *Computer Vision and Image Understanding*.

[40] Han KS, Lim JH, Im EG Malware analysis method using visualization of binary files.

